# The N14 anti-afamin antibody Fab: a rare V_L_1 CDR glycosylation, crystallographic re-sequencing, molecular plasticity and conservative *versus* enthusiastic modelling

**DOI:** 10.1107/S205979831601723X

**Published:** 2016-11-29

**Authors:** Andreas Naschberger, Barbara G. Fürnrohr, Tihana Lenac Rovis, Suzana Malic, Klaus Scheffzek, Hans Dieplinger, Bernhard Rupp

**Affiliations:** a Division of Biological Chemistry, Medical University of Innsbruck, Innrain 80, 6020 Innsbruck, Austria; b Center for Proteomics, University of Rijeka, B. Branchetta 20, 51000 Rijeka, Croatia; c Division of Genetic Epidemiology, Medical University of Innsbruck, Schöpfstrasse 41, 6020 Innsbruck, Austria; d Vitateq Biotechnology GmbH, Innrain 66, 6020 Innsbruck, Austria; e CVMO, k.-k. Hofkristallamt, 991 Audrey Place, Vista, CA 92084, USA

**Keywords:** antibody fragment, flexibility, variable-chain glycosylation, elbow angle, precision, accuracy, solvent, non-apparent isomorphism, solvent modelling

## Abstract

The monoclonal antibody N14 is used as a detection antibody in ELISA kits for the human glycoprotein afamin, a member of the albumin family, which has recently gained interest in the capture and stabilization of Wnt signalling proteins, and for its role in metabolic syndrome and papillary thyroid carcinoma. As a rare occurrence, the N14 Fab is N-glycosylated at Asn26L at the onset of the V_L_1 antigen-binding loop, with the α-1–6 core fucosylated complex glycan facing out of the L1 complementarity-determining region. The crystal structures of two non-apparent (pseudo) isomorphous crystals of the N14 Fab were analyzed, which differ significantly in the elbow angles, thereby cautioning against the overinterpretation of domain movements upon antigen binding. In addition, the map quality at 1.9 Å resolution was sufficient to crystallographically re-sequence the variable V_L_ and V_H_ domains and to detect discrepancies in the hybridoma-derived sequence. Finally, a conservatively refined parsimonious model is presented and its statistics are compared with those from a less conservatively built model that has been modelled more enthusiastically. Improvements to the PDB validation reports affecting ligands, clashscore and buried surface calculations are suggested.

## Abstract 2

Models of the V_L_1 glycosylated Fab fragment independently refined from two non-apparent (pseudo) isomorphous crystals show significant differences, allowing the meaning of accuracy in structure description to be revisited, while at the same time inviting reflections about the benefits and boundaries of complex solvent modelling and validation.

## 1. Introduction   

### 1.1. The N14 monoclonal antibody: function and unique features of its antibody fragment   

Horseradish peroxidase-conjugated murine N14 IgG1κ monoclonal antibody (mAB) is the detecting antibody in a novel sandwich ELISA used for quantification of the human glycoprotein afamin (Dieplinger *et al.*, 2013 ▸; Dieplinger & Dieplinger, 2015 ▸), a plasma vitamin E-binding glycoprotein of the albumin gene family (Voegele *et al.*, 2002 ▸). Afamin (AFM) is a biomarker for metabolic syndrome and related cardiovascular disease as well as for ovarian cancer (Dieplinger *et al.*, 2009 ▸; Kronenberg *et al.*, 2014 ▸; Seeber *et al.*, 2014 ▸). Strong interest in the AFM crystal structure results from the fact that it seems to be, at least *in vitro*, a carrier for Wnt signalling proteins (which are relevant in cell proliferation pathways), which are otherwise very hard to solubilize and to purify (Mihara *et al.*, 2016 ▸). A potential role of afamin in the glucose metabolism in papillary thyroid carcinoma has been reported (Shen *et al.*, 2016 ▸), and the N14 Fab (fragment, antigen binding) can serve as a scaffolding partner in AFM crystallization. The N14 Fab displays a number of interesting structural features and its crystallization in two crystal forms with non-apparent isomorphism also allows an extended analysis of its structural flexibility and of the practice and effects of extensive solvent model building.

### 1.2. Variable-domain IgG glycosylation   

In addition to the frequent and in part conserved glycans of antibody Fc (fragment, crystallizable) domains (Arnold *et al.*, 2007 ▸), glycosylations in the Fab regions of IgG antibodies emerging primarily through somatic hypermutation have gained increasing interest owing to their influence on IgG function and immune regulation (van de Bovenkamp *et al.*, 2016 ▸). Genomic cDNA analysis reveals that about 15–25% of Fabs are expected to be glycosylated overall (Anumula, 2012 ▸), while only ∼9% of the variable regions are glycosylated based on genomic cDNA analysis (Arnold *et al.*, 2007 ▸). Glycosyl­ations in the variable regions that are functionally relevant to antigen (Ag) binding, for example, have been described at Asn58H, Asn60H and Asn54H (Gala & Morrison, 2004 ▸). We report crystallographic evidence for a rare glycosylation at Asn26L at the onset of the variable light-chain L1 loop (V_L_1) of the complementarity-determining region (CDR). Additional instances of variable-chain glycosylations of largely unknown function detected in PDB models are compiled in Supplementary Table S1 (see §[Sec sec3.3]3.3).

### 1.3. Crystallographic sequence verification   

In order to successfully patent an antibody, various claims are stated, with the most common being the sequence (Holliday, 2009 ▸). With the decreasing cost of genomic sequencing, the sequences of the V_H_ and V_L_ domains (or of the set of six CDRs) started to dominate. To avoid competition, but also to prevent a threat from subsequently detected deviations from the patented sequences, as was the case for N14, patent claim rules usually permit changes in the CDR sequences provided that 90 or 95% sequence identity is retained (van der Hoff, 2014 ▸). One of the most important items is to show that the claimed antibody is an alternative to known antibodies. The existence of glycans within the variable domains can then become a valuable piece of information in supporting the claim. This is particularly the case owing to the emerging importance of IgG Fab glycosylation in the immune response (van de Bovenkamp *et al.*, 2016 ▸).

The variable-region sequences of antibodies are most frequently determined *via* RNA extraction from hybridoma cells, reverse transcription, PCR and cDNA sequencing or *via* mass-spectroscopic methods (see Zhang *et al.*, 2014 ▸). Crystallographic model building allows the sequence to be verified, serving as a powerful alternative complementing these techniques. Given a sufficiently high resolution (better than ∼2 Å), the shape of the reconstructed electron density and the chemical environment of side chains is expected to conform to expectations. At sufficient map quality and resolution, however, difference electron density and implausible stereochemistry can indicate sequence discrepancies. We were able to correct three sequence assignments, emphasizing the benefit of careful inspection of difference maps, and affirming the value of mAb variable-region sequence-propensity compilations (Wu & Kabat, 1970 ▸; Martin, 1996 ▸).

### 1.4. One model might not be enough   

The accuracy and precision of a molecular-structure model represent two different qualities. The precision of individual atomic coordinates, which for small-molecule structures is directly obtainable from the covariance matrix (Sheldrick & Schneider, 1997 ▸), is rarely computed in biomolecular refinement (Tickle *et al.*, 1998 ▸), largely because the inversion of the Hessian second-derivative matrix is computationally too expensive for highly multiparametric models (Tronrud, 2004 ▸). Instead, estimated global measures such as the diffraction precision index (DPI; Cruickshank, 1999 ▸) or measures derived from maximum-likelihood (ML) estimates are substituted (Vagin *et al.*, 2004 ▸). Higher resolution in general provides a larger amount of data and a correspondingly smaller variance or higher precision of atomic coordinates upon refinement. In contrast, the accuracy of macromolecular structures is not clearly defined. While from a purely statistical viewpoint, accuracy can be interpreted as the deviation of the expected mean from an unknown true value (reflecting systematic errors), *macromolecular accuracy* is a context-sensitive and less well defined quality: in different environments, biological macromolecules can crystallize in different crystal structures, and altered packing contacts can capture different conformational states. Various means of the visualization of such conformational variance within a set of crystal structure models have been suggested (see Kantardjieff *et al.*, 2002 ▸). The N14 Fab fragment provides an excellent example where independent refinement of related Fab crystal structures leads to models with significant conformational local and long-range differences.

### 1.5. Conservative *versus* ‘enthusiastic’ model   

As of yet, no real consensus exists in the structural biology community as to up to which point weak electron density should be modelled (Read & Kleywegt, 2009 ▸). Interpreting weak density can often be ambiguous, but even at low electron-density levels the interpretation of features based on reasonable prior expectations such as known solvent composition or consensus about expected glycosylations can be considered to be plausible. Overly enthusiastic interpretation, however, often results in poor local real-space correlation, excessive *B* factors and poor stereochemistry in the low-density regions. As a result of the high *B* factors and/or partial occupancies reducing the X-ray scattering contribution, only small differences in global reciprocal-space statistics such as *R* values appear. Statistical *R*-value-based Hamilton tests (Hamilton, 1965 ▸) or likelihood or Bayes ratio tests (Kass & Raftery, 1995 ▸) exist, but they are rarely used or, given the small differences, do not always allow conclusive answers about which model is better. We therefore elected to deposit both a conservative model and an ‘enthusiastic’ model of one of the N14 Fab structures and suggest some practical points for maintaining a parsimonious model without unduly restricting experienced model building.

### 1.6. Fab-domain notation   

The Kabat notation (Wu & Kabat, 1970 ▸) assigned by *AbNum* from the *KabatMan* suite (Martin, 1996 ▸) is applied throughout the manuscript for N14 residue numbering. The two different chains of the antibody fragment are assigned as L (light chain) and H (heavy chain). Each Fab chain consists of a variable domain (V_L_ and V_H_, respectively) harbouring the six complementarity-determining regions (CDRs) and a constant domain (C_L_ and C_H_1, respectively); the domain boundaries in Kabat notation are defined as V_L_ ≤ L107 < C_L_ and V_H_ ≤ H113 < C_H_1.

## 2. Experimental   

### 2.1. Antibody and Fab preparation   

Monoclonal antibodies against human afamin (N13 and N14) were obtained with conventional hybridoma technology (Köhler & Milstein, 1975 ▸) by immunizing BALB/c mice with purified human afamin dissolved in phosphate-buffered saline solution (PBS) pH 7.4, as described in the supplemental material of Dieplinger *et al.* (2013 ▸). Affinity-purified mouse monoclonal IgG1κ antibodies were concentrated to 2 mg ml^−1^ in PBS and cleaved into Fab and Fc fragments according to the protocol of Andrew & Titus (2001 ▸). In brief, the antibodies (2 mg ml^−1^ in PBS) were dissolved in equal volumes of freshly prepared 2× digestion buffer (0.035 *M* EDTA, 40 m*M* l-cysteine in PBS). Papain (0.1 mg ml^−1^) was also freshly prepared in 2× digestion buffer and equal volumes of antibody and papain were mixed and incubated (37°C, 2 h). The reaction was stopped by adding iodo­acetamide to a final concentration of 30 m*M*. Fab fragments were separated from Fc fragments and remaining uncleaved IgG on an ÄKTA FPLC equipped with a Protein A column. The Fab fragments from the flowthrough were concentrated in PBS using centrifugal filter concentrators (molecular-weight cutoff 10 kDa). Papain was removed by size-exclusion chromatography (SEC) using a Superdex 200 10/300 column on an ÄKTApurifier 100 FPLC system (SEC buffer; 20 m*M* HEPES pH 7.5, 150 m*M* NaCl). The Fab solution was concentrated with a centrifugal filter concentrator (Vivaspin VS2021, 30 kDa cutoff) to a final concentration of 10 mg ml^−1^. The purity of the Fab was assessed by Coomassie-stained SDS–PAGE analysis.

### 2.2. Sequence determination   

The genomic sequence of the variable domains (V_L_ and V_H_) was determined by Oak Biosciences, Sunnyvale, California, USA *via* RNA extraction from hybridoma cells, reverse transcription, PCR and cDNA sequencing (http://www.oakbiosciences.com/). Subsequent to the discovery of three discrepancies between electron density and the assigned genomic sequence, mass-spectrometric MALDI-TOF peptide mapping of the N14 Fab at the Protein Micro-Analysis Facility, Medical University of Innsbruck with 98% sequence coverage of the V_L_ chain and 80% coverage of the V_H_ chain was performed (see §[Sec sec3.2]3.2).

### 2.3. Crystallization   

Crystals were grown at 291 K by sitting-drop vapour-diffusion in 96-well plates (Swissci 30926) using 200 nl droplets of antibody-fragment stock solution (10 mg ml^−1^ SEC-purified Fab fragment in 20 m*M* HEPES pH 7.5, 150 m*M* NaCl) mixed with 200 nl crystallization cocktail in a robotic setup using a Phoenix robot (Art Robbins Instruments, Sunnyvale, California, USA) equipped with a single nanoneedle protein dispenser (Krupka *et al.*, 2002 ▸; Naschberger *et al.*, 2015 ▸). Block-shaped crystals with sharp edges (0.15 × 0.15 × 0.3 mm) grew within a day without optimization from the Wizard PEG Ion 1 screen (Rigaku Reagents) in conditions A3 [N14A3; 30% polyethylene glycol mean molecular weight 1 kDa (PEG 1K) and 200 m*M* ammonium fluoride pH 5.5] and C3 (N14C3; 30% PEG 1K, 200 m*M* potassium fluoride pH 5.8).

### 2.4. Data collection   

Crystals were manually harvested using suitably sized MiTeGen cryo-loops and cryo-meshes mounted on bar-coded SPINE standard bases, and were flash-cooled without additional cryoprotection. The pins were placed in SPINE pucks and transferred in dry shipping dewars to beamline ID29 at the ESRF (de Sanctis *et al.*, 2012 ▸) for robotic crystal mounting. Diffraction data were collected at 100 K in single-wavelength mode at 1.0000 Å (12.398 keV) using a Dectris PILATUS 6M detector in fine-slicing mode from automatically pre-screened crystals using the *mxCuBE* beamline-control software (Gabadinho *et al.*, 2010 ▸). Data were processed by the *EDNA* automated data-processing pipeline (Monaco *et al.*, 2013 ▸) employing *XDS* and *XSCALE* (Kabsch, 2010 ▸), *POINTLESS*, *AIMLESS* and *CTRUNCATE* from the *CCP*4 program suite (Winn *et al.*, 2011 ▸) and *phenix.xtriage* from the *PHENIX* suite (Adams *et al.*, 2011 ▸). To exclude any effects of possible isomorphism between the two data sets biasing *R*_free_, the cross-validation flags from the N14A3 data (1.86 Å resolution) were transferred to N14C3 (1.88 Å resolution). Conservative CC_1/2_ cutoffs of 0.69 and 0.47, respectively, were selected for the last resolution shells (Karplus & Diederichs, 2012 ▸; Diederichs & Karplus, 2013 ▸); the remaining data statistics are listed in Table 1 ▸.

### 2.5. Structure determination   

#### 2.5.1. Automated molecular replacement and *ARP*/*wARP* model building   

The merged and unique data set of structure factors together with the separate light-chain (L) and heavy-chain (H) sequences of murine Fab 12E8 (Trakhanov *et al.*, 1999 ▸) were submitted to CCP4 Online (http://www.ccp4.ac.uk/ccp4online) for processing with the *BALBES* automated structure-solution pipeline (Long *et al.*, 2008 ▸). In both cases the best solution was obtained with an assembly of chains 1IL1(A)+1IL1(B) (Berry *et al.*, 2001 ▸). The molecular-replacement model was then automatically submitted to *ARP*/*wARP* 7.5 (Langer *et al.*, 2008 ▸) and the resulting models were manually rebuilt with *Coot* (Emsley *et al.*, 2010 ▸) and refined with *REFMAC*5 (Murshudov *et al.*, 2011 ▸) from the *CCP*4 suite v.6.5 (Winn *et al.*, 2011 ▸) using the *CCP*4*i* graphical interface (Potterton *et al.*, 2003 ▸).

#### 2.5.2. Manual model building and refinement   

**Figure 1:**
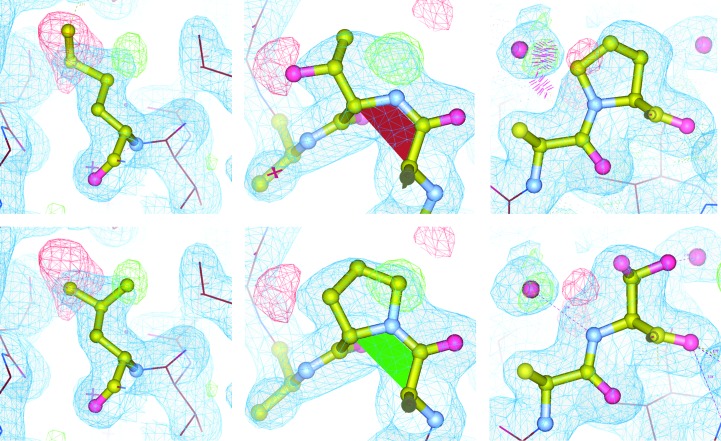
Sequence corrections to the N14 model. The top row shows the originally assigned sequences (Met4L, Thr8L and Pro84L) and the row below the corrected side chains (Leu4L, Pro8L and the split Ser84H in two conformations). The ball-and-stick models are displayed in 2*mF*_o_ − *DF*_c_ electron density displayed at 1σ (blue grid) and *mF*_o_ − *DF*_c_ difference density (2.8σ; green and red grid for positive and negative difference density, respectively) after refinement of the original N14C3 model (Met4L, Thr8L and Pro84H) with *REFMAC* (Murshudov *et al.*, 2011 ▸) and are rendered in *Coot* (Emsley *et al.*, 2010 ▸). Note how the unusual non-Pro *cis*-peptide indicated by the red plane indicator reverts to a common pre-Pro *cis* conformation (green) and how the incorrectly sequenced ProH84 causes a clash (red spikes) with, and displaces, the adjacent water atom, which is also consistent with the difference density. The atom contacts were calculated with the *MolProbity* suite (Chen *et al.*, 2010 ▸).

Repeated cycles of manual rebuilding in real space assigning the commercially determined V_L_ and V_H_ domain sequences and the germline sequences of the murine BALB/c constant chains, followed by restrained reciprocal-space refinement with *REFMAC* (Murshudov *et al.*, 2011 ▸) using the default flat masked solvent model, led to final models of good stereochemical quality after constrained group occupancy refinement of alternate conformations (Table 2 ▸). Inspection of difference density maps in N14C3 as well as N14A3 revealed three sequence discrepancies in the V_H_ and V_L_ domains (§[Sec sec2.2]2.2, Fig. 1 ▸).

After initial automated weight selection, the *REFMAC* Hessian matrix ratio weight was manually optimized to 0.05 by −LL_free_ minimization (Tickle, 2007 ▸) to convergence after the *B*-factor restraint weights were set to empirically determined plausible values (Tronrud, 1996 ▸). A simple Bayes ratio test based on −LL_free_ of the isotropic model without TLS (serving as a null hypothesis) and with conservative TLS refinement for separate V_L_ (including the glycan), V_H_, C_L_ and C_H_1 domains (which also appeared plausible by molecular-dynamics TLS analysis; Painter & Merritt, 2006 ▸) did favour the TLS model in the range between ‘positive’ and ‘strongly’ [2ln(*K*) = 7.2; Kass & Raftery, 1995 ▸].

#### 2.5.3. Missing regions of the N14 models   

As commonly observed in Fab crystal structures, several loops at the terminal end of the C_H_1 domain are disordered and are probably present in multiple conformations. Residues in the H127–H133 region in N14C3 had weak and discontinuous electron density, which could not be reliably modelled with a single plausible geometry, and these residues were omitted from the models. Despite the same nominal resolution, the loop regions H127–H133, H155–H163 and H182–H193 as well as the C-terminal residues of both chains are poorly defined in the N14A3 model. Continuous stretches of unidentified branched density in N14A3 which probably originate from missing loop residues could not be modelled. Such density was kept empty and was not filled with water or PEG fragments.

#### 2.5.4. S—S bonds   

The variable-region S—S bonds between H22 and H92 and between L23 and L88 were refined in a single conformation in both N14C3 and N14A3. In N14C3 the constant-region H140–H195 and L134–L194 S—S links were modelled as dual conformers forming two independent S—S links. Additional difference density in both models suggested some radiation damage (Garman, 2010 ▸) at the L134–L194 cysteine link, but no plausible model beyond the occupancy group-refined split Cys–Cys link conformers in N14C3 could be refined.

#### 2.5.5. Glycosylations   

**Figure 2:**
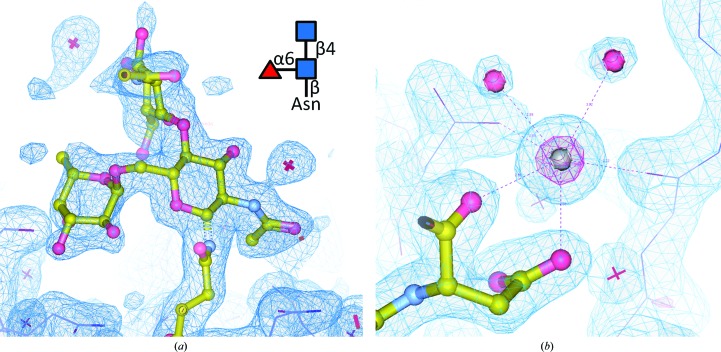
(*a*) Asn26L, located at the onset of L1, points out of the antigen-binding region into solvent. Shown is the first N-linked β-*N*-acetylglucosamine (NAG), the α-1–6-linked fucose and the first β-1–4-linked NAG of the extended glycan branch. 2*mF*_o_ − *DF*_c_ density is displayed at 0.8σ. In the deposited conservative N14 model, only the Asn-linked NAG has been retained. (*b*) The K^+^-binding site linking Asp207H (ball-and-stick representation) and the symmetry-related Ser71H and Asp55H (purple sticks) is shown together with coordinated water molecules (purple balls). Density levels are 1.3σ (blue) and 5σ (magenta, around the K^+^ cation). See §[Sec sec3.5]3.5 for a discussion of glycan modelling.

Asparagine L26, located at the beginning of hypervariable region L1, is glycosylated. A corresponding N-linked *N*-acetylglucosamine (NAG), an α-1–6-linked fucose (FUC) and a β-1–4-linked NAG, pointing out of the antigen-binding region, could be placed into weak electron density in both structures (Fig. 2 ▸ *a*). The modelling of the two branch saccharides (both omitted in the conservatively refined deposited model) is ambiguous (RSCC < 0.7) but is compatible with known Fab glycosylation patterns.

#### 2.5.6. Solvent   

Water molecules were placed individually only into spherical positive difference density of >4.0σ if reasonable contacts to protein or other solvent moieties were present. A few waters were group-refined with correlated partial side-chain occupancies. In N14C3, an initially built water molecule with sixfold coordination and positive difference density despite threefold lower *B* factors compared with the surrounding protein residues was replaced by K^+^, a component of the crystallization cocktail. After completion of model refinement, positive OMIT difference density at the presumed K^+^ site peaked at 16σ. The K^+^ cation mediates a crystal contact between Asp207H and the symmetry-related Ser71H and Asp55H, located in the H2 CDR (Fig. 2 ▸ *b*). Isoelectric Cl^−^ as a stock component (§[Sec sec2.1]2.1) was deemed to be less plausible because the site is highly negatively charged. Consistent with the absence of K^+^ from the cocktail components, the corresponding site in N14A3 is less defined and was modelled with a water molecule. Given a somewhat lower *B* factor for this water than the average neighbour *B* factor, a mixture of water with additional unknown metal ions and/or ammonium ions from the cocktail cannot be excluded. Bivalent cations such as Ca^2+^ and Mg^2+^ are less likely owing to their significantly shorter coordination distances. The entire contact region in N14A3 is less ordered than in N14C3, with some weak positive difference density fragments remaining in the solvent.

Numerous stretches of unbranched continuous (difference) electron density were interpreted as ordered fragments of polyethylene glycol [PEG; HO(CH_2_CH_2_O)_*n*_H] from the crystallization cocktail. A number of remaining positive difference density fragments in the disordered terminal regions, in the vicinity of the unmodelled flexible loops and in disconnected density in the solvent regions could not be plausibly modelled.

#### 2.5.7. Validation   

Refinement parameters and restraint weights are listed in Table 2 ▸ and (redundantly) in the PDB header, and validation reports are available from the PDB. The top and only serious close contacts reported are not valid because *MolProbity* (Davis *et al.*, 2007 ▸; Chen *et al.*, 2010 ▸) as implemented by the PDB does not recognize partial correlated occupancies constrained to 1.0 in the absence of ALTLOC identifiers. The sole Ramachandran outlier reported by *Coot*, Ser51L in both N14C3 and N14A3, as well as its neighbouring residues, has an excellent electron-density fit (RSCC > 0.9) and the backbone geometry must therefore be considered as supported by evidence. Ser51L is located in a conserved γ-turn and is frequently observed in a high-energy conformation (Stanfield *et al.*, 2006 ▸). *RAMPAGE* (Lovell *et al.*, 2003 ▸) and the PDB validation reported this residue in an allowed region. The single K^+^ ion in N14C3 was validated and cross-checked using *CheckMyMetal* (Zheng *et al.*, 2014 ▸). The conformations of the refined glycan anomers were validated using *Privateer-validate* (Agirre *et al.*, 2015 ▸) and agreed with expectations. Elbow angles were calculated with the *RBOW* Fab elbow-angle web service (Stanfield *et al.*, 2006 ▸). Additional validation criteria relevant to the discussion of model differences are provided in Table 2 ▸.

#### 2.5.8. Modelling differences   

The ‘enthusiastically’ built model of N14C3 was obtained by successively adding model features to the conservative N14C3 starting model. During these steps, TLS parameters were kept constant and no restraint weight optimization was conducted. After the final additions, TLS parameters were again refined, with the matrix weight remaining at 0.05. Details of and the motivation for the extension of the model are provided in §3.5 *ff*.

## 3. Results and discussion   

### 3.1. Unit-cell metric   

The relation between the two crystal structures N14A3 and N14C3 is different from what the lattice metric suggests at a first glance. When the unit-cell parameters and reflection indices are ordered by the convention^1^ *a* < *b* < *c*, the models are not related by expected crystallographic transformations. The cell has expanded (about 5% in volume) so that the new *a* in N14A3 is now longer than *b*. To bring the models into an isomorphous setting, the reflections of the original N14A3 *a* < *b* < *c* cell needed to be re-indexed as *k*, *h*, −*l*, and the model needed to be transformed with the Cartesian transformation (*x*, *y*, *z*) = (*y* + 1/4*a*, *x* + 1/4*b*, −*z* + 1/4*c*), with *a* > *b* < *c* for the new cell. The relation between these two cells is best described as non-apparent isomorphism.^2^ We therefore deposited two models of N14A3, one in the setting conforming to the *a* < *b* < *c* convention (PDB entry 5l7x) and one in the swapped cell setting (PDB entry 5lgh) so that the models can directly be displayed within their properly related unit cells. All TLS records and anisotropy tensors have been converted to the new setting.

### 3.2. Crystallographic sequence assignment   

Three sequence discrepancies associated with a single codon change were detected during model building, with the side chains as identified by electron-density (mis)match being highly plausible given the corresponding variable-region sequence propensities (Martin, 1996 ▸; Johnson & Wu, 2000 ▸). Residue Met4L (ATG) with a Kabat probability (KP) of 0.55 was unambiguously identified from electron density as Leu (CTG) with a KP of 0.41; residue Thr8L (ACA) with a KP of 0.07 was unambiguously identified from electron density as Pro (CCA) with a KP of 0.9. Residue Pro84H (ACT), with a KP of 0.07, was identified from electron density with high probability as a Ser in a split conformation (TCT), with a KP of 0.39. Thr and Ala as alternative possibilities (KPs of 0.11 and 0.25) to Pro84H generated negative and positive difference density, respectively, and were deemed to be less plaus­ible (Fig. 1 ▸). Posterior mass-spectrometric MALDI-TOF peptide mapping of the N14 Fab at the Protein Micro-Analysis Facility, Innsbruck Medical University, with 98% sequence coverage of the V_L_ chain and 80% coverage of the V_H_ chain, identified all peptide fragments of N14 sequence as assigned by electron-density inspection.

### 3.3. Differences between the two N14 crystal structures   

The two structure models refined against data from non-apparent isomorphous crystals obtained from the same batch of protein stock under identical setup conditions with a difference in the cation in the 200 m*M* cocktail additive (NH_4_F *versus* KF) and associated pH changes diverge significantly in tertiary structure (domain conformation) as well as in local details. The N14C3 model with 5% lower unit-cell volume is of higher overall quality, with a better map appearance and fewer disordered regions than N14A3, despite comparable data-quality statistics and resolution. The variable-domain backbones differ between the two structures slightly more than with coordinate precision (ΔV_L_ 0.170 Å, ΔV_H_ 0.191 Å), while the differences between the constant-domain backbones are significantly larger (ΔC_L_ 0.336 Å, ΔC_H_1 0.348 Å).

Detailed analysis of the intermolecular contacts using *PISA* (Krissinel & Henrick, 2007 ▸) reveals that the K^+^ metal-binding site exhibits a high complexation significance score (CSS), indicating that the formation of this intermolecular interface involving six residues is likely to be a prominent factor in the tighter packing of the N14C3 crystal form. Transition-metal ions in particular are frequently included in crystallization cocktails as intermolecular contact-promoting additives (McPherson, 1982 ▸; Trakhanov *et al.*, 1998 ▸).

#### 3.3.1. Elbow angles   

**Figure 3:**
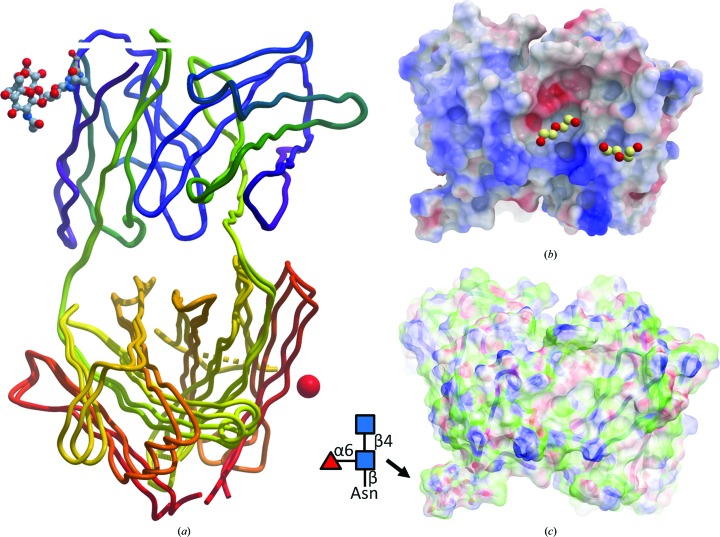
Overall structure of the N14 anti-afamin antibody Fab fragment. (*a*) provides an overview of the N14A3 and N14C3 structure models superimposed on the V_L_ and V_H_ domains. The different domain orientation of the constant regions is distinctly recognizable, with the larger elbow angle of N14A3 pushing the C_L_ and C_H_ domains wider apart, which may be one contributing factor to the 5% larger unit-cell volume of N14A3. The backbone traces are shown as tube models coloured from the N-terminus (dark blue) to the C-terminus (red), with the location of the K^+^-binding site in N14C3 indicated by a red sphere and the Asn26L glycosylation shown as a ball-and-stick model. (*b*) Electrostatic surface presentation (blue, positive charge; red, negative charge) of the antigen-binding region (including the glycan, bottom left). The deep cleft between the V_L_ and V_H_ chains in the centre of the solvent-exposed CDR region contains a PEG fragment embedded in a discrete water network (not shown). The second PEG fragment to the right mediates a crystal contact. The displayed PEG fragments are present in both structures. (*c*) Surface map of binding properties: white, neutral surface; green, hydrophobic surface; red, hydrogen-bonding acceptor potential; blue, hydrogen-bond donor potential. In (*b*) and (*c*) the glycan branch can be seen protruding at the bottom left. Surface calculations and figure rendering by MolSoft *ICM Browser Pro* (Abagyan *et al.*, 1994 ▸).

The elbow angles of the two N14 Fab models differ significantly, by 8°, with the more open N14A3 form packing less densely. Both elbow angles are close to the mode of the rather broad elbow-angle distribution typical for the κ-chain IgG antibodies (Stanfield *et al.*, 2006 ▸). The wider elbow angle in N14A3 increases the distance between the C_L_ and C_H_ domains compared with N14C3 (Fig. 3 ▸), which is consistent with the larger unit-cell volume and smaller buried L–H contact surface of the N14A3 *versus* N14C3 crystal form (Table 2 ▸).

In antibody Fab fragment structure refinement, differences in elbow angles have been observed even between different NCS-related copies in the same crystal (Stanfield *et al.*, 1990 ▸, 2006 ▸), sometimes with elbow-angle changes exceeding 20° (PDB entry 1jnh, 27° difference; PDB entry 1s78, 22° difference; PDB entry 1ots, 21° difference). Given that molecular-dynamics simulations of Fab-domain movement predict hinge-bending fluctuations with only 2–3° r.m.s.d. in elbow angle in solution (Sotriffer *et al.*, 2000 ▸), the significant differences in elbow-angle change between the N14A3 and N14C3 Fab models are almost certainly a consequence of the different crystallization conditions. While the antibody community has learned to exercise caution when assigning significance to antibody-domain rearrangements, strong conclusions about domain orientations from a single-crystal structure may be a risky proposition if not supported by independent assessment of the solution conformation or multiple crystal structures (Kantardjieff *et al.*, 2002 ▸).

### 3.4. Antigen-binding site and glycosylation   

The antigen-binding site projects almost entirely into intermolecular solvent, with exception of the loops affected by a crystal contact (Table 3 ▸). The deep antigen-binding cleft between the V_L_ and V_H_ chains is occupied by a PEG fragment embedded in a water network in both structures. Interestingly, the glycosylation of Asn26L was observed (but was not further expanded on) in an early milestone paper on mAB–antigen peptide binding (Stanfield *et al.*, 1990 ▸), with the site sequence Asn26L-Gln27L-Thr27(A)L in an extended L1 CDR loop.

The Asn26L-Ser27L-Ser27(A)L sequence is the only N-glycosylation site present in N14, and the prior probability of observing the N-glycosylation consensus site sequence Asn26L-Xxx27L-(Ser,Thr)27(A)L in a IgG1-κ mouse Fab is quite low. Under the assumption of independence, the prior probability based on Kabat propensities that a glycosylation at position 26L occurs is *P*(glyc|26) = *P*(Asn|26) × *P*(Ser,Thr|27A) = 0.009 × (0.906 + 0.016) = 0.008; that is, about 1 in 100 mouse IgG1-κ Fab models with an insertion at position 27(A) are expected to present this feature at site 26L. In IgG1κ Fabs with no 27L insertions, the corresponding probability is about 0.2%. Exposed glycans on IgG variable loops tend to be complex and fully sialylated (Arnold *et al.*, 2007 ▸).

**Figure 4:**
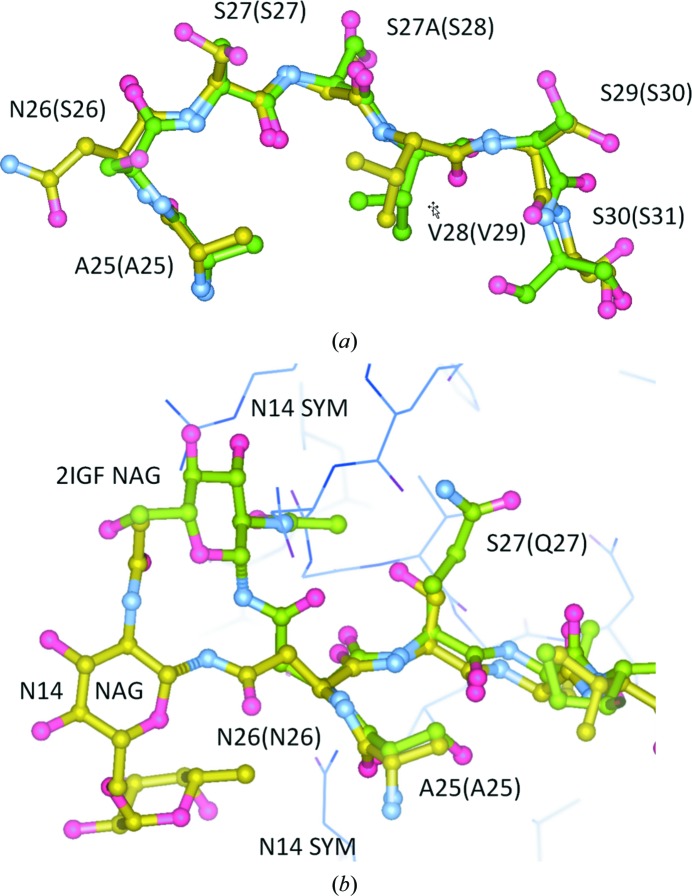
L1 CDR and glycan-binding site. (*a*) Main-chain superposition of N14 L1 CDR (yellow sticks) with the ‘canonical conformation structure 1’ (green sticks; PDB entry 2fbj; Al-Lazikani *et al.*, 1997 ▸). The glycosylation at Asn26L located at the onset of the hypervariable region has little effect on the canonical L1 conformation. (*b*) The glycan fragments in N14 (yellow sticks) and PDB entry 2igf (green sticks; Stanfield *et al.*, 1990 ▸) cannot possibly assume the same conformation despite a very similar loop arrangement around Asn26L, because a symmetry-related molecule in N14 (thin blue sticks) would interfere with the 2igf glycan conformation in the case of N14. This figure was displayed and rendered with *Coot* (Emsley *et al.*, 2010 ▸).

The N14 L1 loop harbouring the N-glycosylation site reveals almost an identical conformation to the ‘canonical structure 1’ defined by Al-Lazikani *et al.* (1997 ▸), with the exception of a peptide-bond flip at position Ser29L-Ser30L, distant from Asn26L (Fig. 4 ▸ *a*). A comparison between the N-linked NAG glycan in PDB entry 1igf (where it could be modelled in only one of the two NCS-related copies of the unbound Fab fragment) and PDB entry 2igf (Fab bound with Ag peptide) with the N14 conformation shows that while the glycan does not seem to directly participate in peptide antigen binding in PDB entry 2igf (Stanfield *et al.*, 1990 ▸), the glycan conformation is clearly affected by the packing of neighbouring molecules, while the canonical CDR conformation is maintained (Fig. 4 ▸ *b*). Given that Asn26L is located at the onset of hypervariable region L1 but pointing out from the antigen-binding region, with little effect on the canonical L1 conformation, crystallographic evidence for a functional role of the glycosylation may become available based on an AFM–N14 complex crystal structure.

A simple text search of the PDB for antibody models containing N-glycans identified five different IgG Fabs with V_L_ chain glycosylations [sites Asn22, 25, 26(2×) and 72] and eight instances with V_H_ glycosylations (31, 52, 55, 57, 72, 73, 88, 96), most of them containing only one or two modelled NAG saccharides. A spreadsheet containing these instances (among almost 200 search results with NAG moieties in other parts of the model or complex) is deposited as Supplementary Table S1.

### 3.5. Examining the trade-off between parsimony and interpretative freedom   

The steady improvement in structural model-validation tools and the flagging of questionable models by the community have led to increasing scrutiny of structure models. Better structure models will improve the quality of the research that is based on them, and enable more reliable meta analysis and data mining of structure repositories (Dauter *et al.*, 2014 ▸). As a consequence of the trend towards improved validation, almost all journals now realise the importance of providing at least PDB validation reports to reviewers (see Fink, 2016 ▸). Examining these reports can certainly prevent grossly flawed models (which have previously escaped detection) entering the literature and becoming persistent in the PDB (Rupp *et al.*, 2016 ▸). However, the sole reliance on PDB validation reports is not always sufficient to judge the validity of claims, because the desire to provide simple metrics for structure quality does not do justice to the complex task of local model evaluation. Inspection of electron density is *de facto* necessary for the full analysis and review of a structure model. In addition, given the difficult mandate of the PDB Validation Task Force (Read *et al.*, 2011 ▸) to cover almost every conceivable aspect of model validation, constant improvement of the reports to eliminate errors and ambiguities, or to reconsider metrics that cannot be applied to each and every situation, are desirable.

A particularly intense feature of the validation reports are the outlier reports, which are highlighted to draw the attention of the depositor (or the ire of the reviewer). To explore the ability of a reasonably experienced crystallographer and the restraint necessary to produce a reasonably ‘clean’ PDB validation report, we refined and deposited the N14C3 model optimistically (meaning that we extended our interpretative freedom to lower density levels while at the same time not introducing obviously conjectural or wrong model features) and with more parsimonious restraints, attempting to obtain a validation report with a reasonably achievable minimum of outliers. Examining these models might help aspiring model builders to develop their own level of comfort for the in­evitable compromise between reflective restraint and excessive modelling enthusiasm.

#### 3.5.1. The degree of surprise   

Outliers are not necessarily or always errors. They are expected to occur with a defined frequency given by the amount of deviation of their respective statistic from the sample mean or their deviation from an empirical distribution. An intuitive way to look at them is to judge them by how much they surprise us. Surprise is directly related to informational entropy (Stone, 2015 ▸) and is a powerful aid in judging the relevance of an outlier. A bond-length outlier with a 5σ deviation (RMSZ = 5, with an expected frequency of occurrence of ∼1/150 000) does surprise us and is, pending further investigation, very probably an error. Whether a Ramachandran outlier surprises us depends on conditioning information: without supporting electron density it is likely to be a simple modelling error and our surprise is modest, while supporting clear electron density turns it from an outlier into an interesting feature worthy of further contemplation.

### 3.6. Remarks on modelling practice   

#### 3.6.1. Backbone and close contacts   

Given the representative resolution of 1.9 Å (the PDB mean is around 2.2 Å), the models were not allowed to have unsupported Ramachandran outliers and only few close contacts (clashes; Table 2 ▸) with a deviation of less than ∼0.6 Å. Large deviations from prior expectations in general either need correspondingly strong evidence to be considered plausible, or most likely are real errors that should be corrected, irrespective of any allowances for interpretative freedom. The true clashscore for all models is 1 (less than 1 in 1000 contacts). All Asn, Gln and His side-chain flips proposed by *MolProbity* were also examined at this stage. Except for one ambiguous suggestion involving a symmetry-related molecule in PDB entry 5l7x, all Asn, Gln and His side-chain orientations could already be assigned correctly during model building based on forming the most plausible hydrogen-bond networks.

It is important to realise that the φ and ψ backbone torsion angles are normally not restrained in reciprocal-space refinement and provide valuable geometric cross-validation. With the protein backbone being one continuous chain and the bond lengths and bond angles highly restrained, the only option that the refinement program has to reduce scattering contributions from the model in places where the data do not justify this is to increase the *B* factors and/or to move the atoms to places where they are less compromising in the overall refinement target. The unrestrained backbone torsion angles allow and absorb such movements and the resulting outliers indicate that the model is not plausible in its current conformation. While correcting the model to energetically favourable backbone torsion angles in *real space* is reasonable, restraining the backbone torsion in *reciprocal-space refinement* is permissible only in rare circumstances. Low resolution in general provides additional opportunities for modelling errors and risky interpretation. The 2008 and 2011 CCP4 Study Weekend proceedings compiled in the February 2009 and April 2012 issues of *Acta Crystallographica Section D* contain key references on refinement and validation of low-resolution crystal structures.

The PDB validation report employs *RAMPAGE* (Lovell *et al.*, 2003 ▸), with more permissible allowed backbone torsion-angle regions than the more restrictive values of Kleywegt & Jones (1996 ▸) that are actually listed in the PDB file header under REMARK 500.

#### 3.6.2. Water   

Water molecules were manually placed with environmental restraints in mind. Irrespective of the absolute density level, the density needed to be reasonably spherical and plausible contacts to protein or neighbouring solvent molecules had to be present. Placing waters, particularly early on, into continuous blobs of significant density that are obviously not just water does slightly improve the global refinement statistics, but carries the penalty of obscuring the shape of the surrounding difference density. This difference density generally improves in shape as the refinement progresses, and a more cognizant decision as to the nature of the unknown moiety might then be made.

#### 3.6.3. Disordered or missing model parts   

Crystallo­graphers tend to take personal affront when parts of a structure cannot be modelled because no supporting density exists. In most circumstances (aside from proteolytic cleavages and related instances) one is reasonably sure that the absent part does exist, but its location is not defined. Although this is normal. as many solution structures have demonstrated, it irks the eager model builder, and *some* model is built, absent of convincing density. Such enthusiasm bears two consequences. Punishment is generally promptly delivered in the form of poor local real-space statistics, combined with implausible geometry, and the futile circle of rebuilding and obtaining frustrating results is repeated *ad tedium*.

Secondly, placing a model in a specific conformation (or two) when a whole ensemble of models might be equally plausible can create a problem for unsuspecting users. Although the resulting high *B* factors make localized electron density disappear in agreement with reality, the atom records in the PDB, and thus the balls and sticks in graphical representations, firmly remain. Zero occupancies assigned to questionable atoms are an equally imperfect remedy, if not recognized by the user. An ensemble model with its multiple chains occupied in accordance with their prior stereochemical probabilities and given local environment restraints would probably come closest to reality. Such composite models and the means and ways of their representations (Koradi *et al.*, 1996 ▸) are a matter of fact in NMR structure studies and have been applied to macromolecules (Kantardjieff *et al.*, 2002 ▸). Four residues in the disordered C_H_1 domain (A129H, A130H, Q131H and T132H) and two terminal residues (D1L and C215H), as well as one ambiguous split side chain, are not included in the conservative model, while the optimistic model lacks only A130H and Q131H and D1L (see also Table 2 ▸).

#### 3.6.4. Glycans   

In many cases, N-linked glycan decorations are only partly visible, with their electron density deteriorating towards the solvent-exposed branch. Low-level density indicates that a chain of covalently linked sugars is present, but likely in multiple and dynamically changing conformations. Placing a model there on one hand does make it clear that something has to be there, meaning that this region is almost certainly excluded from access by other parts of the molecule or by its complex partners. This ‘excluded volume’ is *de facto* valuable information, for example in complex modelling, but one single specific model does not reflect the real situation. The punishment for placing in part justified but ambiguous models is again poor density fit, possibly geometry violations, and a high, yellow-highlighted LLDF because the glycans are considered ‘ligands’. The arguable compromise we selected is to show the actual density and a possible but not unique model in Figs. 2 ▸ and 3 ▸, demonstrating that something has to be there, but depositing in the conservative model only the positional and conformationally certain Asn-linked NAG and omitting the second NAG also in the enthusiastic model. While simple omission is the easiest way to keep the PDB report ‘clean’, one has to realise that such a practice does not do justice to reality. How to adequately present such situations of ‘known absences’ remains an open question.

#### 3.6.5. PEG fragments   

**Figure 5:**
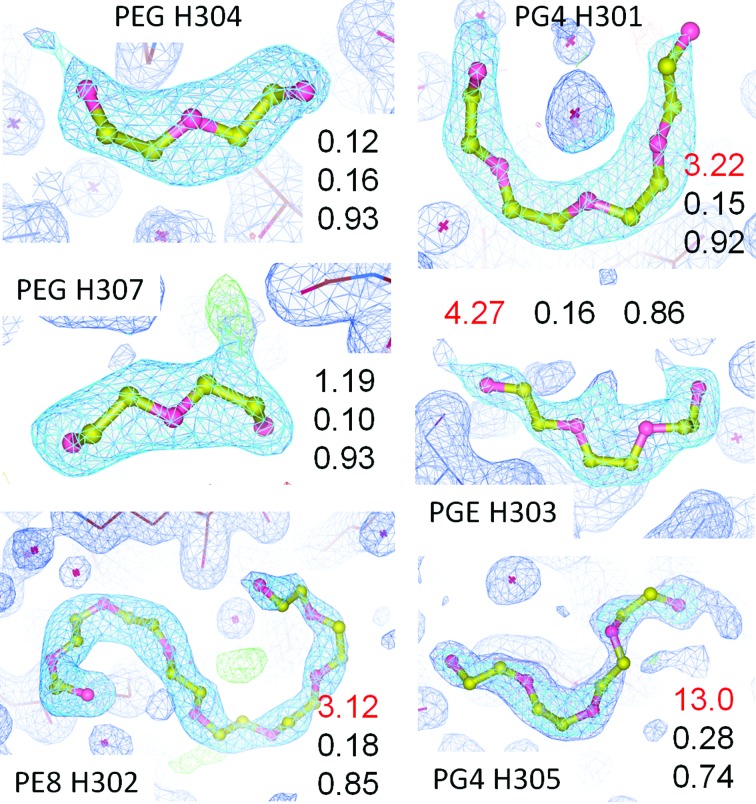
PEG fragments ranked by LLDF. The PEG fragments are labelled as numbered in PDB entry 5l6d and are ranked by the LLDF (best to worst), which is the first item in the data columns/row, followed by the real-space *R* value (RSR) and the real-space correlation coefficient (RSCC) as listed in the validation report. Density levels are 1σ for the 2*mF*_o_ − *DF*_c_ map (blue grid, highlighted around the PEG fragments) except for PG4 H305, where the contours of the blue map have been lowered to 0.70σ. Difference density is shown at ±3σ levels. Red LLDF numbers correspond to yellow highlights in the validation report. The reason for the scores is not always transparent, which emphasizes the need for electron-density inspection for ligands and caution against simple acceptance (or condemnation) based on the LLDF score. For example, in PEG H307 it is obvious that the model is insufficient to explain the additional density (perhaps an alternate conformation), while the very plausible hydrogen-bond network of PG4 H301 does not prevent a highlighted (presumably bad) score. Note that in contrast to H301 the central positive density peak in PE8 H302 could not be modelled with a water molecule because it was impossible (or beyond our limit of tedium) to orient the PE8 in one unique conformation where only O atoms would form a reasonable hydrogen-bond network to the neighbouring molecules. Incorrect register of the O *versus* C atoms frequently causes improper close contacts to neighbouring atoms. 2*mF*_o_–*DF*_c_ density is displayed at 1.2σ. Note also that the assumption that PEG fragment models must always end with a terminal oxygen (–OH) at each end is an unrealistic expectation. This figure was displayed and rendered with *Coot* (Emsley *et al.*, 2010 ▸).

PEGs [polyethylene glycol, polyethylene oxide, H(OCH_2_CH_2_)_*n*_-OH] and their monomethyl ethers (PEG MMEs) are the most frequently used precipitants in crystallization trials (McPherson, 1976 ▸, 1985 ▸). Despite their apparent structural (but certainly not chemical; see Ray & Puvathingal, 1985 ▸) simplicity, they are difficult to model correctly: almost always only ambiguous fragments are identifiable [even low-molecular-weight PEG 400 has about nine (OCH_2_CH_2_) oxyethylene repeats on average]. Only when the hydrogen bonds to the environment are well defined can a decision be made whether a C atom or an O atom should be placed at a given location (see PG4 H301 in Fig. 5 ▸). More often than not, bad contacts rather than neat hydrogen bonds indicate a less offending register of O and C atoms in the PEG fragment chains. In addition, the present practice of assigning a specific chemical entity based on the modelled fragment length and perceived terminal atom is unrealistic. Table 4 ▸ lists the PDB identifiers of available PEG fragments of 1 ≤ *n* ≤ 14 as a (at present) useful reference during model building. A more systematic family tree of PEG fragments with consistent and easily extensible (restraint file) nomenclature would be desirable.

PEG chains also have a similar shape (but considerably more conformational freedom) than peptide backbones. Before building multiple conformations or instances of PEGs to model branched continuous density, plausible explanations such as an ordered piece of an otherwise missing peptide loop or terminus should be considered. Premature solvent placement can also obscure the shape of difference density. An additional seven PEG fragments have been added to the seven PEG fragments of the conservative model, and only two fragments (see Fig. 5 ▸), conserved between the structures, passed the muster of the LLDF validation metric.

### 3.7. Lessons learned: less can be more, but is it more accurate?   

The comparison of the different models of the same crystal structure refined with different modelling philosophies allows some interesting and also concerning conclusions. A fundamental principle which we applied to all models is that no obvious errors were accepted, and interpretative freedom was limited to parts in weak or ambiguous density, including solvent.

Statistics and parameters relevant to the comparison are listed in Table 2 ▸. Firstly, the removal of protein features placed in weak density, such as terminal residues or residues flanking chain breaks or of PEG molecules in very low density, has a limited effect on the reciprocal-space statistics: the optimistic model has lower *R* values, with Bayes ratios indicating ‘somewhat to positively better’, while the *R*–*R*_free_ gap perhaps indicates marginally less overmodelling for the conservative model. Consistent with this modest assessment is the effect of the relative *R*_free_ decrease: *R*_free_ is lower by 1.9%, which is comparable to the estimated precision (∼1.7%) of *R*_free_ (Tickle *et al.*, 2000 ▸). Only small effects on global reciprocal statistics are expected because the high *B* factors reduce the already small scattering contribution of the few ambiguous model parts even further. Small increases in real-space outliers and side-chain torsion outliers are attributable to the presence of more ambiguous protein residues in the less parsimonious model. The conclusion here is that if the model is reasonable to begin with, a few low-density residues more or less will not significantly affect the overall model statistics, but their inclusion or absence can affect the accuracy of the model in practical terms. The value of whether a questionable residue or decoration is modelled or not depends on the intended use of the model: is it for example important to know that a certain region is excluded from interactions or is perhaps not surface accessible, or that a glycan is likely to cause steric hindrances?

The increase in modelled PEG fragments does have some interesting consequences. Only few, even plausibly modelled, fragments can satisfy the LLDF criteria. We are not convinced that eliminating most PEGs to satisfy the PDB validation report is meaningful, and we suggest that solvent entities and flexible decorations such as glycans are not judged by the justifiably stricter criteria for a *bona fide* bound ligand. A rather concerning result is that the buried surface area between the two protein chains as reported by the automated use of *PISA* (Krissinel & Henrick, 2007 ▸) for the PDB file annotation does correlate strongly with the number of modelled PEG fragments (see the recommendations below).

The effective trade-off between the attempt to achieve completeness of a structure model by including parts modelled with low confidence, at the risk of introducing stereochemistry violations, has also surfaced in a comparison of structural genomics (SG) initiative models with non-SG models. The largely automatically built SG models tend to be more conservatively built, but less complete, than crystal structure models deposited by the general structural biology community (Read & Kleywegt, 2009 ▸).

#### 3.7.1. Suggestions for improving PDB reports and structure annotation   

PDB validation reports are undoubtedly helpful for detecting and correcting previously missed, late-stage errors in models. In particular, correcting clashes (improbable close contacts) and examining backbone angle and rotamer outliers clearly improves the posterior probability of the model, as such errors are frequently also shown by careful difference density inspection (for model correction, lower contours than the ‘default’ 3σ difference density are often informative). However, some legitimate modelling is also flagged as errors. Given the fact that these reports are increasingly issued as a basis for review, we hope that the remaining issues that we have identified can be addressed.

(i) The concurrent use of backbone-angle analysis programs with different boundaries delivers inconsistent results. The actual PDB files still include outliers reported using the 1996 metrics (Kleywegt & Jones, 1996 ▸) in REMARK 500, while the *RAMPAGE* (Lovell *et al.*, 2003 ▸) results in the validation report show no such outliers. This discrepancy is simply confusing and reporting should be made consistent.
(ii) A series of erroneously reported serious clashes in PDB entries 5l9d and 5l88 can be traced back to problems that *MolProbity* (Chen *et al.*, 2010 ▸) presently has in correctly interpreting correlated group occupancies when one of the members does not have an alternate conformation. This is the case, for example, when a water molecule is present at occupancy *n* and the conflicting conformation of a split residue is occupied at 1 − *n*. We also found a partially occupied Met whose terminal –CH_3_ group apparently conflicted with a neighbouring residue 3.3 Å away. In this instance, no H atoms were calculated for one of the alternate conformations and we have no explanation for the origin of this problem. The corresponding *REFMAC* group occupancy keyword file is supplied as Supporting Information.
(iii) Complex solvent components other than water are the greatest obstacle to obtaining a clean PDB report. In view of the reports of ligand models without density support, the well intended LLDF quality metric makes sense in structure models where a ligand is proposed to be tightly bound to a molecule in a specific pose, serving to support some biological hypothesis: a strong claim indeed requires a strong proof. Solvent molecules are currently classified as ligands, and in the case of conformationally promiscuous PEG fragments or glycan decorations, the stringent LLDF criteria highlight as suspect even models at defensible RSCCs of as high as 0.9 (Fig. 5 ▸). Eliminating such molecules from the model simply to satisfy a presently too broadly applied stringent metric is not necessarily the best option. The area occupied by glycans, for example, is excluded from access, which is potentially useful and valid information. Low-density and omit difference density contours do carry some information in those instances. How to model such entitles at the solvent boundary (see Holton *et al.*, 2014 ▸; Weichenberger *et al.*, 2015 ▸), doing justice to their ambiguity while not categorically condemning those attempts at obtaining a more accurate model, has not yet been resolved.
(iv) Table 2 ▸ reveals that while the solvent-accessible surface area (reported automatically in the PDB file by *PISA*; Krissinel & Henrick, 2007 ▸) between the Fab complex formed between the L and H chains remains almost constant, the buried surface area increases by nearly 50% when more PEG molecules are added to the model. This is obviously an artefact which cautions that these values as listed in the PDB file greatly depend on the quality and extent of solvent modelling. If such differences are possible even between different instances of the same structure model, a comparison between different structure models becomes completely dependent on individual solvent modelling and corresponding caution in the interpretation of the automatically created values as listed in the PDB REMARK 350 header is advisable.

The origin of the discrepancies in the *PISA* calculation seems to come from the very unfortunate diktat of the PDB to override author assignment of solvent moieties such as PEGs by changing the chain IDs to that of the nearest protein chain and assigning the ‘ligand’ category to it. Without manually turning off the perceived PEG ligands in *PISA*, the buried surface area between chains thus becomes a function of the extent of solvent modelling. While glycans are a *bona fide* component of the macromolecule itself, the solvent molecules originating from crystallization precipitants are decidedly not and are highly variable depending on the environmental context.

## 4. Concluding remarks   

The two independently refined crystal structure models of the N14 anti-afamin antibody Fab fragment crystallizing in two crystal forms with non-apparent isomorphism allowed a number of interesting observations. N14 serves as the detection antibody in afamin ELISAs (Dieplinger *et al.*, 2013 ▸), and its Fab antibody fragment can be used for crystallization scaffolding experiments of the complex glycosylated human plasma protein afamin, which is a promiscuous transporter of hydrophobic molecules, including vitamin E (Voegele *et al.*, 2002 ▸). Afamin may also play a role in the Wnt signalling pathway or serve as a chaperone enhancing the solubility of Wnt proteins (Mihara *et al.*, 2016 ▸) and has a potential role in glucose metabolism in papillary thyroid carcinoma (Shen *et al.*, 2016 ▸).

Electron density at 1.9 Å resolution in combination with prior expectations based on antibody sequence (Kabat) variability was sufficient to detect three clear sequencing discrepancies probably caused by single codon-read errors. The V_L_ and V_H_ sequences were obtained from commercial sequencing of the BALB/c hybridoma cell lines used for mAB production, and verification by independent means such as mass-spectrometric sequence mapping or high-resolution crystallographic studies may be prudent.

The deposition of two different models, one conservatively refined in an attempt to minimize outliers in the PDB validation reports and the other optimistically interpreted, show that is not possible to satisfy PDB reports without losing some valid information that could be relevant for users of the model. It is important that reviewers are made aware of how to interpret the reports in these contentious areas and that ultimately only the inspection of electron density can provide clarity about the validity of claims based on X-ray crystallo­graphic studies. After all, the scientists must be the judges of their hypotheses and not the (validation) statistician (Edwards, 1992 ▸).

## Supplementary Material

PDB reference: N14 Fab, crystal form I, parsimonious model, 5l9d

PDB reference: crystal form I, non-parsimonious model, 5l88

PDB reference: crystal form II, 5l7x

PDB reference: crystal form II: same as 5l7x, but isomorphous setting indexed the same as 5l88 and 5l9d, 5lgh

Supplementary Table S1. Variable-chain glycosylations in PDB antibody-fragment models.. DOI: 10.1107/S205979831601723X/rr5136sup1.xlsx

Group occupancy keyword file for REFMAC.. DOI: 10.1107/S205979831601723X/rr5136sup2.txt

## Figures and Tables

**Table 1:** Crystallization, data collection and structure solution

Crystal (PDB entries)	N14C3 (5l9d, 5l88)	N14A3 (5lgh, 5l7x)
Stock solution	10 mg ml^−1^ N14 Fab in 20 m*M* HEPES pH 7.5, 150 m*M* NaCl	
Crystallization conditions	30% PEG 1K, 0.2 *M* KF pH 5.8	30% PEG 1K, 0.2 *M* NH_4_F pH 5.5
ESRF ID29 wavelength (Å)	1.0000	1.0000
ESRF data identification	lat-N14_615_C3_w1_run1	lat-N14_615_A3_w1_run2
Space group (No.)	*P*2_1_2_1_2_1_ (19)	*P*2_1_2_1_2_1_ (19)
Unit-cell parameters† (Å)	*a* = 67.78 (9), *b* = 69.25 (8), *c* = 87.80 (9)	*a* = 72.20 (3), *b* = 67.49 (5), *c* = 88.94 (6) (5lgh)
Non-isomorphous setting	N/A	*a* = 67.49 (5), *b* = 72.20 (3), *c* = 88.94 (6) (5l7x)
Unit-cell volume† (Å^3^)	412113 (1401)	433385 (797)
Solvent fraction	0.439	0.466
*V*_M_ (Å^3^ Da^−1^)	2.19	2.31
Wilson *B* factor (Å^2^)	36.6	40.0
Resolution‡ (Å)	48.44–1.88 (1.95–1.88)	44.47–1.86 (1.93–1.86)
Completeness‡ (%)	99.4 (97.7)	98.6 (91.3)
Observed reflections‡	154121 (10340)	142315 (9822)
Average redundancy‡	4.5 (3.2)	3.9 (3.0)
〈*I*/σ(*I*)〉‡	10.0 (1.4)	8.8 (1.0)
*R*_meas_ ‡ § (%)	9.1 (91.4)	9.5 (114.3)
*R*_merge_ ‡ ¶ (%)	7.6 (80.4)	7.4 (93.4)
CC_1/2_ ‡ †† (%)	99.8 (68.8)	99.8 (46.6)
*BALBES* results		
*Q*-score	0.814	0.815
*R*_free_	0.297	0.298
*R*	0.337	0.339
Δ*R*_free_	0.152	0.170

† Values in parentheses are estimated standard uncertainties of the last significant digit(s).

‡ Values in parentheses are for the highest resolution shell.

§ *R*_meas_ = 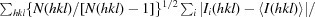 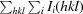, where *I*_*i*_(*hkl*) is the *i*th of *N*(*hkl*) observations of reflection *hkl* and 〈*I*(*hkl*)〉 is the weighted average intensity for all observations of reflection *hkl* without symmetry merging.

¶ *R*_merge_ = 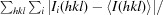 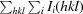, where *I*_*i*_(*hkl*) is the *i*th observation of reflection *hkl* and 〈*I*(*hkl*)〉 is the weighted average intensity for all symmetry-merged (unique) observations of reflection *hkl*.

†† CC_1/2_ is Pearson’s correlation coefficient between two randomly assigned data sets each derived by averaging half of the observations for a given reflection.

**Table 2:** Refinement, validation and analysis of the deposited models The table contains statistics of interest for model comparison in §§[Sec sec3.2] [Sec sec3.3] [Sec sec3.4]3.2–3.4. The PDB headers include the full information.

Model	N14C3, conservative	N14C3, optimistic	N14A3
Refinement			
PDB entry	5l9d	5l88	5l7x (5lgh)
*R*_free_ (5% set)	0.215	0.211	0.228
*R*_work_	0.176	0.170	0.190
Δ*R*	0.039	0.041	0.038
TLS groups	4 (V_H_, V_L_ + glycan, C_H_1, C_L_)	4 (V_H_, V_L_ + glycan, C_H_1, C_L_)	4 (V_H_, V_L_ + glycan, C_H_1, C_L_)
No. of atoms			
Protein	3280	3300	3224
‘Ligand’	97	189	64
Waters	221	252	223
All refined non-H	3598	3741	3511
〈*B*〉 (Å^2^)			
Protein atoms	33.5	35.6	39.0
‘Ligand’ atoms	61.5	74.4	59.2
Waters	40.0	41.6	41.5
All refined non-H atoms	34.7	38.0	39.5
Refined occupancy groups	12	13	4
Glycans	Asn26L-NAG	Asn26L-NAG-FUC	Asn26L-NAG
PEG fragments	7	14	5
Missing residues	6	3	16
Coordinate errors (Å)			
Free	0.139	0.140	0.139
σ_A_	0.119	0.120	0.141
Cruickshank DPI	0.154	0.156	0.154
*F*_o_ *versus* *F*_c_ correlation	0.967	0.970	0.968
*F*_o_ *versus* *F*_c_ correlation, free	0.956	0.956	0.955
R.m.s.d., bond lengths (Å)	0.009	0.009	0.011
R.m.s.d., angles (°)	1.36	1.37	1.50
Geometry			
Elbow angle (°)	139	139	147
Clashes, true, reported	4 of 10	5 of 11	3 of 3
Ramachandran†			
Total	431	436	412
Allowed	7	8	6
Outliers	0	0	0
Real-space *R* outliers, RSRZ > 2	8	9	10
Side-chain rotamer outliers	6	9	14
Buried contact surface, reported, L+H‡ (Å^2^)	6350, 1834	9640, 1854	5250, 1700
LLDF ‘ligand’ outliers	5 of 9	10 of 17	5 of 6

† As determined using the Ramachandran plot boundaries by Lovell *et al.* (2003 ▸).

‡ L+H signifies the actual surface contact area between protein residues of the L and H chains, obtained by excluding the solvent molecules from the contact calculation (*cf.* the output of the detailed contact tables provided by the *PISA* web service at http://www.ebi.ac.uk/pdbe/pisa/).

**Table 3:** Sequences of the six hypervariable loops of the complementarity-determining regions (CDRs) of the afamin-binding region depicted in Fig. 2 ▸ Highlights are explained in the remarks column.

CDR region	Residues	Remarks
L1 (L24–L34)	TA**N**SSVSSNYFH	Asn26L glycosylated
Canonical L1	-ASSSVSS-	
L2 (L50–L56)	ST**S**NLAS	Ser51L in high-energy conformation
L3 (L89–L97)	HQYHRSPPT	
H1 (H31–H35)	SYIIH	
H2 (H50–H65)	YINPYN**D**GSKYNEKFKG	Asp55H in K^+^-mediated crystal contact
H3 (H95–H102)	NYWSDSLDY	

**Table 4:** List of PDB three-letter identifiers (PDB codes) for PEG and PEG MME fragments with *n* ≤ 14 Many additional and inconsistently named fragments of presumably the same chemical entities exist, terminating with two C atoms (examples are 16P, 7PE, AE4, AE3, P3G, PE4, PE5 and others).

No. of PEG units (*n*)	PEG PDB ID C_2*n*_O_*n*+1_H_4*n*+2_ HO(CH_2_-CH_2_-O)_*n*_H	PEG MME PDB ID C_2*n*+1_O_*n*+1_H_4*n*+4_ HO(CH_2_-CH_2_-O)_*n*_CH_3_
1	EDO	MXE
2	PEG	PG0
3	PGE	TOE
4	PG4	ETE
5	1PE	1PG
6	P6G	P15
7	P33	—
8	PE8	7PG
9	2PE	—
10	XPE	—
11	—	—
12	12P	—
13	33O	—
14	PE3	—

## Footnotes

1 This convention is in fact unfortunate, because it leads to proliferation of different axis permutations of the same space group. In space group 18, *P*2_1_2_1_2, for example, a swap of *a* with *c* would require deposition in space group *P*22_1_2_1_.

2 We thank Zbigniew Dauter, ANL for this suggestion.
